# Effectiveness of pirfenidone in idiopathic pulmonary fibrosis according to the autoantibody status: a retrospective cohort study

**DOI:** 10.1186/s12890-021-01516-4

**Published:** 2021-05-03

**Authors:** Myung Jin Song, Sang Hoon Lee, Ji Ye Jung, Young Ae Kang, Moo Suk Park, Young Sam Kim, Joon Chang, Song Yee Kim

**Affiliations:** 1 Division of Pulmonary and Critical Care Medicine, Department of Internal Medicine, Seoul National University College of Medicine, Seoul National University Bundang Hospital, Seongnam, Republic of Korea; 2 Division of Pulmonology and Critical Care Medicine, Department of Internal Medicine, Severance Hospital, Yonsei University College of Medicine, 50-1 Yonsei-ro, Seodaemun-gu, Seoul, 03722 Republic of Korea

**Keywords:** Idiopathic pulmonary fibrosis, Autoantibodies, Pirfenidone

## Abstract

**Background:**

Pirfenidone is an anti-fibrotic agent shown to slow the progression of idiopathic pulmonary fibrosis (IPF). However, its effectiveness in association with serological autoimmune features in IPF remains unclear.

**Methods:**

We retrospectively reviewed the medical records of patients with IPF treated at a tertiary care hospital in South Korea. The autoantibody status was defined as positive if we detected autoantibodies meeting the serological domain criteria for interstitial pneumonia with autoimmune features or anti-neutrophil cytoplasmic antibodies.

**Results:**

We included 142 patients with IPF treated with pirfenidone for over six months (93 were autoantibody-positive and 49 were autoantibody-negative). The mean age was 69.5 ± 7.3 years, and 77.5% of the patients were male. The adjusted mean changes over one year were − 34.4 and − 112.2 mL (*p* = 0.168) in forced vital capacity (FVC), and − 0.53 and − 0.72 mL/mmHg/min (*p* = 0.356) in the lungs diffusion capacity for carbon monoxide (DL_CO_) in the autoantibody-negative and autoantibody-positive groups, respectively.

**Conclusions:**

Reductions in FVC and DL_CO_ were similar in autoantibody-positive and autoantibody-negative patients with IPF treated with pirfenidone. Pirfenidone is effective in attenuating the progression of IPF, irrespective of the autoantibody status.

## Background

Idiopathic pulmonary fibrosis (IPF) is a chronic, progressive, and fibrosing interstitial pneumonia of unknown cause, characterized by a histopathological pattern of usual interstitial pneumonia (UIP) [1]. Exclusion of known causes of interstitial pneumonia, including environmental exposures, drug toxicities, and connective tissue diseases (CTDs), is important for IPF diagnosis because it affects the treatment and prognosis [2, 3].

Rheumatoid arthritis, systemic lupus erythematosus, idiopathic inflammatory myopathies, Sjögren’s syndrome, systemic sclerosis, and mixed connective tissue disease are representative CTDs that might involve the lungs and cause fibrotic lung disorders. Each CTD has its unique clinical features and presents specific autoantibody positivity that can help to distinguish it from other CTDs. An official IPF diagnostic guideline proposed by the International Consensus Statement of the American Thoracic Society and European Respiratory Society also recommends serologic autoantibody tests in all patients with newly identified interstitial lung disease to exclude CTDs [1]. However, CTDs might initially involve only the lungs, without extrathoracic features [4]. Furthermore, symptoms and signs of extrapulmonary involvement might not be present at the time of diagnosis, or they might be subtle [5]. These factors complicate the differential diagnoses of fibrotic lung disease.

The term interstitial pneumonia with autoimmune features (IPAF) was recently proposed to describe individuals with interstitial lung disease and other clinical, serologic, and morphologic features that presumably arise from an underlying autoimmune condition but do not meet the current diagnostic criteria for a CTD [6]. This group of patients demonstrates better survival than patients with IPF but markedly worse survival than those with CTD-related interstitial lung disease [7, 8]. The diagnostic criteria of IPAF include the clinical, serologic, and morphologic domains.

However, UIP is excluded from the morphologic domain because its association with CTD is weaker than that of the morphologic patterns observed in other diseases, such as non-specific interstitial pneumonia, organizing pneumonia, and lymphoid interstitial pneumonia. Therefore, patients with UIP, positive for autoantibodies, who do not meet the diagnostic criteria for CTD, are diagnosed with IPF and treated according to the IPF guidelines.

Autoantibody positivity has previously been reported in 23–41% of patients with IPF [9–13]. To date, little is known about the clinical implications of autoantibody positivity in IPF, and the reported results are somewhat controversial. Some studies reported that autoantibody positivity was associated with a better survival outcome [10, 12], while others reported no survival difference between autoantibody-positive and autoantibody-negative patients [11, 13]. One retrospective study reported that treating autoantibody-positive IPF with immunomodulators was associated with a superior survival outcome [12]. However, it is not known if IPF with different serological autoimmune presentation respond differently to the antifibrotic treatment.

This retrospective study aimed to investigate whether pirfenidone, an antifibrotic agent currently used to treat patients with IPF, has differential effects in patients with different autoantibody statuses.

## Methods

### Patients

**Fig. 1:**
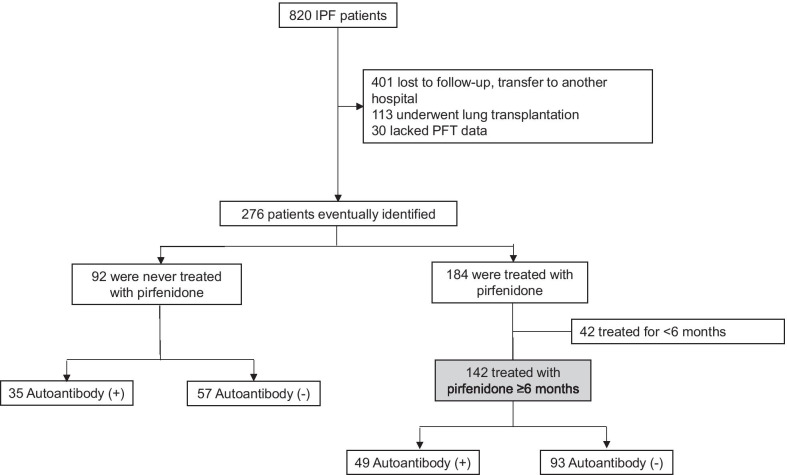
Patient recruitment flow chart. *IPF* idiopathic pulmonary fibrosis, *PFT* pulmonary function test

We retrospectively reviewed the medical records of patients with IPF treated at Severance Hospital, a tertiary care university hospital in South Korea, between January 2013 and March 2018. A total of 820 patients of IPF were initially screened, and 544 were excluded for the following reasons: lost to follow-up or transfer to another hospital (n = 401), underwent lung transplantation (n = 113), absence of two or more pulmonary function tests (PFTs) separated by at least six months (n = 30). After excluding those patients, 276 patients were eventually identified. Of these, 92 were never treated with pirfenidone and 184 were treated with pirfenidone, and 142 of them treated for more than 6 months. The patient recruitment flowchart is presented in Fig. 1. The Institutional Review Board and Ethics Committee of Severance Hospital approved this study (number: 4-2018-0435). All procedures were performed in accordance with the Declaration of Helsinki. The requirement for written informed consent was waived due to the retrospective study design.

### Definitions

IPF was diagnosed by a multidisciplinary team of pulmonologists, radiologists, and pathologists specializing in chest diseases. The diagnosis was based on the diagnostic criteria set by the International Consensus Statement of the American Thoracic Society and European Respiratory Society in 2011 [14]. The autoantibody status was considered positive if the serologic test detected any of the screened autoantibodies in the IPAF serologic domain [8] or anti-neutrophil cytoplasmic antibodies (ANCA).

### Serologic autoantibodies analysis

The following serologic autoantibodies were evaluated: antinuclear antibody (ANA), rheumatoid factor (RF), cyclic citrullinated peptide antibody, anti-dsDNA antibody, anti-Ro antibody, anti-La antibody, anti-ribonucleoprotein antibody, anti-Smith antibody, anti-Scl-70 antibody, anti-Jo-1 antibody, myeloperoxidase, ANCA, and proteinase-3 ANCA. ANA positivity with a diffuse, homogeneous, or speckled staining pattern and a titer cutoff value of 1:320 was required to classify the test as positive [15]. ANA was considered positive irrespective of the titer if accompanied by either a nucleolar or centromere staining pattern. Serum RF level greater than or equal to twice the normal upper limit was classified as positive. We set these minimums because low ANA and RF titers are present in some patients without rheumatic autoimmune disorders and even healthy individuals [16, 17]. Other circulating autoantibodies with any value above the normal upper limit were considered positive.

### Statistical analysis

The forced vital capacity (FVC) and diffusing capacity of the lungs for carbon monoxide (DL_CO_) were compared between baseline and 12 months later using the linear mixed model with Bonferroni correction. Corrections for age, sex, height, and weight, all of which affect the FVC, were included in the linear mixed model. Continuous variables were analyzed by the Student’s *t*-test and Wilcoxon signed rank-sum test. Categorical variables were analyzed by the Chi-square and Fisher’s exact tests. In all cases, differences with a *p-*value < 0.05 were considered statistically significant. Statistical analyses were performed using the R statistical software, Version 4.0.0 (R Foundation for Statistical Computing, Vienna, Austria).

## Results

### Patient characteristics

Ninety-two patients who were never treated with pirfenidone and 142 patients who received pirfenidone for over six months were included in this study (Fig. 1; Table 1). Among these 142 patients with IPF, the median follow-up period was 24.4 months (interquartile range, 14.9–32.7 months). The mean age was 69.5 ± 7.3 years, and 77.5% of the patients were male. The patients were divided into two groups according to their autoantibody status: autoantibody-positive (*n* = 93, 65.5%) and autoantibody-negative (*n* = 49, 34.5%). Smoking status, comorbidities, pulmonary function, IPF severity represented by the gender–age–physiology (GAP) index, and the percentage of patients experiencing acute IPF exacerbation during the study period were similar between the two groups. The average pirfenidone dose was also similar between the autoantibody-negative and autoantibody-positive groups (1185.6 and 1183.5 mg, respectively). The proportion of patients using systemic corticosteroids for over 30 days (38.8% vs. 31.2%, *p* = 0.470), and the duration of use (243.0 [125.0–569.0] days vs. 365.0 [140.0–649.0] days, *p* = 0.592) did not show statistically significant difference between the autoantibody-positive group than in the autoantibody-negative group.

**Table 1:** Baseline characteristics of patients

	Patients treated with pirfenidone			Patients never treated with pirfenidone		
	Total (*n* = 142)	Autoantibody ( −) (*n* = 93)	Autoantibody ( +) (*n* = 49)	Total (*n* = 92)	Autoantibody ( −) (*n* = 57)	Autoantibody ( +) (*n* = 35)
Sex (male)	110 (77.5%)	71 (76.3%)	39 (79.6%)	64 (69.6%)	40 (70.2%)	724(68.6%)
Age (year)	69.5 ± 7.3	70.0 ± 6.8	68.5 ± 8.0	67.1 ± 10.3	67.1 ± 11.2	67.0 ± 8.7
BMI (kg/m^2^)	24.8 ± 2.9	25.2 ± 3.0	24.0 ± 2.7	23.3 ± 3.0	23.2 ± 3.2	23.5 ± 2.6
Smoking exposure (%)						
Never	43 (30.3%)	32 (34.4%)	11 (22.4%)	7 (7.6%)	4 (7.0%)	3 (8.6%)
Former	80 (56.3%)	50 (53.8%)	30 (61.2%)	51 (55.4%)	34 (59.6%)	17 (48.6%)
Current	19 (13.4%)	11 (11.8%)	8 (16.3%)	34 (37.0%)	19 (33.3%)	15 (42.9%)
Smoking (pack-years)	20.0 (0.0–40.0)	20.0 (0.0–36.0)	30.0 (6.0–45.0)	15.0 (0.0–32.5)	20.0 (0.0–35.0)	4.2 (0.0–30.0)
Comorbidities						
Hypertension	30 (21.1%)	23 (24.7%)	7 (14.3%)	44 (47.8%)	28 (49.1%)	16 (45.7%)
Diabetes mellitus	38 (26.8%)	30 (32.3%)	8 (16.3%)	24 (26.1%)	19 (33.3%)	5 (14.3%)
GERD	46 (32.4%)	30 (32.3%)	16 (32.7%)	27 (29.3%)	19 (33.3%)	8 (22.9%)
Asthma	11 (7.7%)	9 (9.7%)	2 (4.1%)	5 (5.4%)	3 (5.3%)	2 (5.7%)
Old pulmonary tuberculosis	28 (19.7%)	19 (20.4%)	9 (18.4%)	18 (19.6%)	13 (22.8%)	5 (14.3%)
Cancer	29 (20.4%)	18 (19.4%)	11 (22.4%)	23 (25.0%)	17 (29.8%)	6 (17.1%)
Coronary artery disease	25 (19.6%)	14 (15.1%)	11 (22.4%)	19 (20.7%)	11 (19.3%)	8 (22.9%)
Cerebrovascular disease	4 (2.8%)	4 (4.3%)	0 (0.0%)	5 (5.4%)	5 (8.8%)	0 (0.0%)
Pulmonary function test (at IPF diagnosis)						
FVC (L)	2.6 ± 0.7	2.7 ± 0.7	2.6 ± 0.6	2.8 ± 0.9	2.8 ± 0.9	2.8 ± 0.8
FVC % pred	77.0 ± 13.0	78.2 ± 13.9	74.7 ± 10.8	83.6 ± 20.0	81.8 ± 20.6	86.5 ± 18.8
FEV_1_ (L)	2.1 ± 0.5	2.1 ± 0.5	2.1 ± 0.4	2.2 ± 0.7	2.2 ± 0.7	2.2 ± 0.6
FEV_1_% pred	90.7 ± 15.0	91.5 ± 14.8	89.3 ± 15.4	96.2 ± 22.6	94.5 ± 23.5	99.1 ± 21.1
DL_CO_ (mL/mmHg/min)	10.8 (8.7–13.2)	11.1 (8.7–13.6)	9.8 (8.6–12.0)	13.0 ± 4.6	13.4 ± 4.6	12.2 ± 4.5
DL_CO_ % pred	63.0 (53.0–73.0)	65.0 (55.0–76.0)	60.0 (52.0–72.0)	70.5 ± 24.4	72.9 ± 25.9	66.7 ± 21.6
Obstructive pattern	8 (5.6%)	5 (5.4%)	3 (6.1%)	7 (7.6%)	5 (8.8%)	2 (5.7%)
Severity of IPF (GAP index)						
I	126 (88.7%)	81 (87.1%)	45 (91.8%)	48 (84.2%)	48 (84.2%)	32 (91.4%)
II	16 (11.3%)	12 (12.9%)	4 (8.2%)	9 (15.8%)	9 (15.8%)	3 (8.6%)
Follow-up period (months)	24.4 (14.9–32.7)	24.4 (16.6–32.2)	24.2 (11.7–32.7)	31.1 (12.6–54.8)	28.7 (13.8–52.7)	40.3 (12.3–56.0)
Acute exacerbation	26 (18.3%)	20 (21.5%)	6 (12.2%)	13 (14.1%)	10 (17.5%)	3 (8.6%)
Pirfenidone dose (mg)	1184.5 (837.3–1467.3)	1185.6 (890.7–1398.0)	1183.5 (836.0–1550.2)	–	–	–
CT scan pattern^a^						
UIP	124 (87.3%)	86 (92.5%)	38 (77.6%)	76 (82.6%)	48 (84.2%)	28 (80.0%)
Possible UIP	18 (12.7%)	7 (7.5%)	11 (22.4%)	16 (17.4%)	9 (15.8%)	7 (20.0%)
UIP by surgical lung biopsy	44 (31.0%)	26 (28.0%)	18 (36.7%)	30 (32.6%)	18 (31.6%)	12 (34.3%)

Values are expressed as mean ± standard deviation, number (%) or mean (interquartile range)

*BMI* body mass index, *DL*_*CO*_ diffusing capacity of the lungs for carbon monoxide, *FEV*_*1*_ forced expiratory volume in one second, *FVC* forced vital capacity, *GAP* gender, age, and physiology, *GERD* gastroesophageal reflux disease, *IPF* idiopathic pulmonary fibrosis, *IQR* Interquartile range, *UIP* usual interstitial pneumonia

^a^Computed tomography (CT) scan pattern according to the diagnostic criteria set by the International Consensus Statement of the American Thoracic Society and European Respiratory Society in 2011[14]

There was no difference in the proportion of autoimmune positivity according to pirfenidone treatment in all patients with IPF (34.5% vs. 38.0%, *p* = 0.681). The predicted values of FVC (77.0 ± 13.0 vs. 83.6 ± 20.0, p = 0.006), FEV_1_ (90.7 ± 15.0 vs. 96.2 ± 22.6, p = 0.042), and DL_CO_ (63.8 ± 15.8 vs. 70.5 ± 24.4, p = 0.020) in patients treated with pirfenidone were significantly lower than those of patients who were never treated with pirfenidone. This can be explained by the insurance coverage criteria in Korea, as the national insurance covered IPF patients with PFT result of 50% ≤ FVC ≤ 90% and 35% ≤ DLco ≤ 80%. Therefore, patients with very good pulmonary function (FVC ≥ 90% or DLco ≥ 80%) were not treated with pirfenidone.

The positivity rates for the tested autoantibodies are presented in Table 2. The highest observed positivity rate was for ANA, followed by RF.

**Table 2:** Positivity rate for each autoantibody

	Total (*n* = 234)	Patients treated with pirfenidone (*n* = 142)	Patients never treated with pirfenidone (*n* = 92)
ANA	42 (17.9%, *n* = 234)	27 (19.0%, *n* = 142)	15 (16.3%, *n* = 92)
RF	33 (14.4%, *n* = 229)	23 (16.8%, *n* = 137)	10 (10.9%, *n* = 92)
Cyclic citrullinated peptide antibody	8 (3.7%, *n* = 214)	7 (5.7%, *n* = 123)	1(1.1%, *n* = 91)
Anti-dsDNA antibody	3 (2.6%, *n* = 117)	3 (4.0%, *n* = 75)	0 (0.0%, *n* = 42)
Anti-Ro antibody	8 (6.6%, *n* = 121)	4 (5.1%, *n* = 79)	4 (9.5%, *n* = 42)
Anti-La antibody	0 (0.0%, *n* = 121)	0 (0.0%, *n* = 79)	0 (0.0%, *n* = 42)
Anti-ribonucleoprotein antibody	1 (0.9%, *n* = 110)	1 (1.4%, *n* = 71)	0 (0.0%, *n* = 39)
Anti-Smith antibody	2 (1.8%, *n* = 110)	0 (0.0%, *n* = 71)	2 (5.1%, *n* = 39)
Anti-Scl-70 antibody	4 (3.7%, *n* = 109)	0 (0.0%, *n* = 70)	4 (10.3%, *n* = 39)
Anti-Jo-1 antibody	3 (3.0%, *n* = 99)	2 (3.3%, *n* = 60)	1 (2.6%, *n* = 39)
Myeloperoxidase ANCA	31 (13.4%, *n* = 231)	14 (10.1%, *n* = 139)	17(18.5%, *n* = 92)
Proteinase-3 ANCA	7 (3.0%, *n* = 230)	6 (4.3%, *n* = 138)	1(1.1%, *n* = 92)
Autoantibody positive	84 (36.4%)	49 (34.5%)	35 (38.0%)

Values are expressed as number (%, *n* = number of patients in whom antibodies were evaluated)

*ANA* anti-nuclear antibody, *ANCA* anti-neutrophil cytoplasmic antibody, *RF* rheumatoid factor

### Treatment effects

**Fig. 2:**
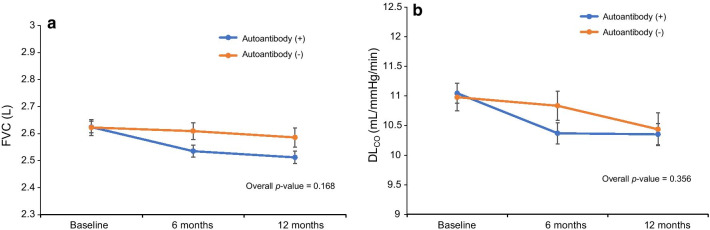
Changes in FVC and DL_CO_ according to the autoantibody status. **a** No difference in the change in FVC between the autoantibody-negative and autoantibody-positive groups (*p* = 0.168); **b** No difference in the change in DL_CO_ between the autoantibody-negative and autoantibody-positive groups (*p* = 0.356). DL_CO_, diffusing capacity of the lungs for carbon monoxide; *FVC* forced vital capacity

The changes in FVC and DL_CO_ were compared between the autoantibody-positive and autoantibody-negative patients treated with pirfenidone. These values were evaluated after adjusting for sex, age, height, weight, and baseline FVC and DL_CO_. The adjusted mean changes in FVC and DL_CO_ over one year did not differ between the two patient groups (FVC: − 34.4 vs. − 112.2 mL, *p* = 0.168; DL_CO_: − 0.53 vs. − 0.72 mL/mmHg/min, *p* = 0.356, for autoantibody-negative vs. autoantibody-positive, respectively; Fig. 2).

**Fig. 3:**
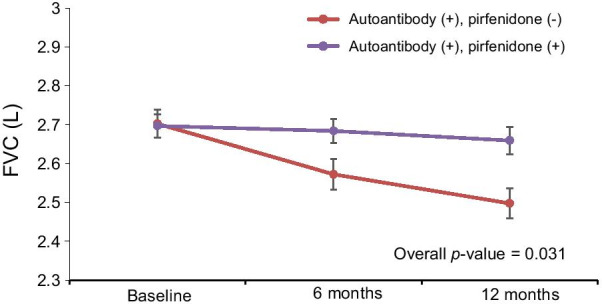
FVC changes in autoantibody-positive patients in relation to pirfenidone treatment. FVC declined in patients who were never treated with pirfenidone more than in those treated with pirfenidone (*p* = 0.031). *FVC* forced vital capacity

Additionally, we compared the adjusted mean FVC changes between autoantibody-positive patients treated with pirfenidone and those who were never treated with pirfenidone. FVC declined more significantly in those who were never treated with pirfenidone than those treated with pirfenidone; the adjusted mean difference in FVC change between these groups was 169.7 mL (*p* = 0.031; Fig. 3).

## Discussion

In this retrospective observational study, we show a similar slowing effect of pirfenidone on the progression of IPF irrespective of the autoantibody status. A similar change in FVC was found in autoantibody-positive and autoantibody-negative patients with IPF. The change in FVC among autoantibody-positive patients treated with pirfenidone was smaller than in those who were never treated with it. Therefore, we conclude that pirfenidone has similar efficacy in attenuating the FVC decline in patients with IPF irrespective of the autoantibody status.

Several studies have reported on the role of autoimmunity in the pathogenesis of IPF. Activated T cells, including autoreactive CD4 T cells, were found in the blood, lung tissues, hilar lymph nodes, and bronchoalveolar lavage fluid of patients with IPF [18, 19]. It was demonstrated that T cells assist B cells in proliferating and producing autoantibodies, and that this process facilitates inflammation and fibrosis in the lungs [20, 21]. However, the clinical implications of this experimentally proven evidence are uncertain.

The rate of autoantibody positivity in patients with IPF in this study was lower than that reported in other studies [12, 13, 22], but similar to that observed in healthy adults [6, 23]. This study was performed at a tertiary university hospital with a medical team specialized in interstitial lung diseases, and only respiratory specialists were authorized to prescribe pirfenidone. Patients diagnosed with IPF and prescribed pirfenidone were routinely followed up by a respiratory specialist. Therefore, although the patients were initially diagnosed with IPF, the respiratory specialist routinely examined them for evidence of alternative diagnoses. Particularly, the autoantibody-positive patients were reevaluated for alternative diagnoses if autoimmune features were observed during follow-up. This might explain the low autoantibody-positive rate in patients with IPF in this study.

Recently, a study reported that autoantibody-positive patients with IPF had a better prognosis than those negative for autoantibodies; additionally, immunomodulators, including steroids, had positive effects on mortality. The authors suggested that different treatment strategies should be enacted based on the presence of autoantibodies [12]. However, even if the autoantibody-positive patients did not meet the autoimmune disease criteria at the time of IPF diagnosis, the close follow-up of true patients with IPF to detect autoimmune diseases demonstrated attenuation of FVC decline due to pirfenidone treatment, an effect that was observed regardless of the autoantibody status. Therefore, we suggest close monitoring of patients with IPF for alternative diagnoses when pirfenidone is ineffective, especially in autoantibody-positive patients, instead of attempting different treatment strategies based on the autoantibody status.

This study has several limitations. First, not all autoantibodies in the IPAF serologic domain were tested in all patients. Therefore, patients included in the autoantibody-negative group may have actually been autoantibody-positive but were not tested for it. Second, masking of the differential effects of pirfenidone due to systemic corticosteroid use cannot be excluded. The proportion of patients using systemic corticosteroids for over 30 days was higher, and the duration of use was longer in the autoantibody-positive group than in the autoantibody-negative group. Although the differences were statistically insignificant, the efficacy difference between the groups could have been masked by the longer and higher systemic corticosteroid use in the autoantibody-positive group. Third, nintedanib, which is another anti-fibrotic approved for the treatment of IPF, was not covered by Korean health insurance until recently [24]. As a result, we were unable to evaluate the impact of autoantibody status on the efficacy of this treatment in our patient population due to small numbers of patients on this medication. Finally, as a single center study, the result of our study requires replication in larger and more diverse patient cohorts to be generalized.

## Conclusions

The patients in this study were diagnosed with IPF after excluding other possible diagnoses. We found that pirfenidone slowed the progression of IPF, independent of the autoantibody status. We suggest that patients with IPF who do not respond to pirfenidone should be reevaluated for underlying autoimmune diseases.

## Data Availability

The datasets used and/or analyzed during the current study are available from the corresponding author on reasonable request.

## Abbreviations

BMI: Body mass index
CT: Computed tomography
DLco: Diffusing capacity of the lungs for carbon monoxide
FEV1: Forced expiratory volume in one second
FVC: Forced vital capacity
GAP: Gender, age, and physiology
GERD: Gastroesophageal reflux disease
IPAF: Interstitial pneumonia with autoimmune features
IPF: Idiopathic pulmonary fibrosis
IQR: Interquartile range
ANA: Antinuclear antibody
ANCA: Anti-neutrophil antibody
RF: Rheumatoid factor
CTD: Connective tissue disease
UIP: Usual interstitial pneumonia
